# Exploring Associations Between Children’s Obesogenic Behaviors and the Local Environment Using Big Data: Development and Evaluation of the Obesity Prevention Dashboard

**DOI:** 10.2196/26290

**Published:** 2021-07-09

**Authors:** Dimitris Filos, Irini Lekka, Vasileios Kilintzis, Leandros Stefanopoulos, Youla Karavidopoulou, Christos Maramis, Christos Diou, Ioannis Sarafis, Vasileios Papapanagiotou, Leonidas Alagialoglou, Ioannis Ioakeimidis, Maria Hassapidou, Evangelia Charmandari, Rachel Heimeier, Grace O'Malley, Shane O’Donnell, Gerardine Doyle, Anastasios Delopoulos, Nicos Maglaveras

**Affiliations:** 1 Lab of Computing, Medical Informatics and Biomedical Imaging Technologies Aristotle University Thessaloniki Greece; 2 Department of Informatics and Telematics Harokopio University of Athens Athens Greece; 3 Multimedia Understanding Group Aristotle University Thessaloniki Greece; 4 Department of Biosciences and Nutrition Karolinska University Stockholm Sweden; 5 International Hellenic University Thessaloniki Greece; 6 Biomedical Research Foundation of the Academy of Athens Athens Greece; 7 Internationella Engelska Skolan Stokholm Sweden; 8 School of Physiotherapy Division of Population Health Sciences RCSI University of Medicine and Health Sciences Dublin Ireland; 9 Insight Centre for Data Analytics University College Dublin Dublin Ireland; 10 College of Business University College Dublin Dublin Ireland

**Keywords:** public health authorities, childhood obesity, children’s behavior, environment, COVID-19, big data, mHealth, uHealth, intervention

## Abstract

**Background:**

Obesity is a major public health problem globally and in Europe. The prevalence of childhood obesity is also soaring. Several parameters of the living environment are contributing to this increase, such as the density of fast food retailers, and thus, preventive health policies against childhood obesity must focus on the environment to which children are exposed. Currently, there are no systems in place to objectively measure the effect of living environment parameters on obesogenic behaviors and obesity. The H2020 project “BigO: Big Data Against Childhood Obesity” aims to tackle childhood obesity by creating new sources of evidence based on big data.

**Objective:**

This paper introduces the Obesity Prevention dashboard (OPdashboard), implemented in the context of BigO, which offers an interactive data platform for the exploration of objective obesity-related behaviors and local environments based on the data recorded using the BigO mHealth (mobile health) app.

**Methods:**

The OPdashboard, which can be accessed on the web, allows for (1) the real-time monitoring of children’s obesogenic behaviors in a city area, (2) the extraction of associations between these behaviors and the local environment, and (3) the evaluation of interventions over time. More than 3700 children from 33 schools and 2 clinics in 5 European cities have been monitored using a custom-made mobile app created to extract behavioral patterns by capturing accelerometer and geolocation data. Online databases were assessed in order to obtain a description of the environment. The dashboard’s functionality was evaluated during a focus group discussion with public health experts.

**Results:**

The preliminary association outcomes in 2 European cities, namely Thessaloniki, Greece, and Stockholm, Sweden, indicated a correlation between children’s eating and physical activity behaviors and the availability of food-related places or sports facilities close to schools. In addition, the OPdashboard was used to assess changes to children’s physical activity levels as a result of the health policies implemented to decelerate the COVID-19 outbreak. The preliminary outcomes of the analysis revealed that in urban areas the decrease in physical activity was statistically significant, while a slight increase was observed in the suburbs. These findings indicate the importance of the availability of open spaces for behavioral change in children. Discussions with public health experts outlined the dashboard’s potential to aid in a better understanding of the interplay between children’s obesogenic behaviors and the environment, and improvements were suggested.

**Conclusions:**

Our analyses serve as an initial investigation using the OPdashboard. Additional factors must be incorporated in order to optimize its use and obtain a clearer understanding of the results. The unique big data that are available through the OPdashboard can lead to the implementation of models that are able to predict population behavior. The OPdashboard can be considered as a tool that will increase our understanding of the underlying factors in childhood obesity and inform the design of regional interventions both for prevention and treatment.

## Introduction

Obesity remains a major health problem worldwide, increasing the risk for the development of noncommunicable diseases, such as diabetes, coronary heart disease, and cancer [1,2]. Its global prevalence has increased dramatically in the last 40 years, resulting in a great economic burden for health care systems [3]. A concerning increase in overweight and obesity among children has also been noted. In 2013, over 42 million children were considered overweight or obese, and approximately 70 million children will be overweight or obese by 2025 [4,5]. In Europe, the prevalence of overweight and obesity in children varies across countries [6], peaking at just over 40% [4,7]. Children with obesity are more likely to be obese until adulthood [8], thereby increasing the risk for chronic disease development [9,10].

Existing evidence indicates that interventions targeting various elements of children’s behavioral patterns, such as *what* and *how they eat* and *how they move* [11], in addition to the living environmental factors to which they are exposed (elsewhere referred to as “ecological” parameters), can yield positive outcomes [8]. To date, the primary sources of evidence for the evaluation of childhood obesity in a given area are self-reporting questionnaires, patient electronic health records, as well as a limited number of focused studies that use objective measurements reflecting children’s behavioral patterns [8].

On a social level, certain environmental factors and socioeconomic parameters have been shown to be associated with obesity in children, such as (1) the ethnicity of the population [12] (pointing to a generalized genetic effect on a population level but mostly toward specific socioeconomic conditions and cultural effects [13], (2) the immediate socioeconomic environment (eg, the “neighborhood” [14], including the immediate proximity to fast food restaurants [15] and the availability of exercise facilities [16]), and (3) exposure to food-related advertising [17].

However, the local environmental context, including the local food environment (eg, the proximity to fast foods), and the local urban characteristics are particularly difficult to quantify, usually requiring extensive, expensive, and long-term studies, making it, until recently, extremely difficult to use those results in a context-based fashion in society.

Fortunately, the widespread adoption and maturation of new technologies, such as the use of mobile phones or smartwatches [18], especially by youth, allow for the collection of a significant volume of information under real-life conditions, which can lead to the extraction of user behavioral patterns [19,20]. Equally, the extensive use of the internet and social media has led to the creation of open databases, such as Google Maps, related to the availability of certain type of places in the local environment. These new technologies, elsewhere referred to as “mobile sensing” [21], open new horizons as a source of objective information. They allow for the more detailed evaluation of environmental effects, not only of the immediate place of residence of the individuals, but also of the areas which they frequent (a data collection challenge previously identified under the term “residential fallacy effect”) [22].

The Big Data Against Childhood Obesity program [23] is a European-funded research project aiming to tackle the problem of childhood obesity using big data collected by children using their smartphones. BigO collects and analyzes big data on children’s behavior and their environment to enable public health authorities to plan and execute effective programs addressing obesity. The BigO system has been developed as a tool for public health authorities to evaluate localized obesogenic behaviors at the population level and to facilitate decision making as well as intervention planning through a powerful analytical framework and a purpose-built dashboard.

This paper presents the Obesity Prevention Dashboard (OPdashboard), which has been developed in the context of the BigO study and can be used to visually explore aggregated children’s localized behaviors and environment characteristics and to automatically extract associations among them. The potential users of the OPdashboard are health researchers in the domains of epidemiology and childhood obesity, as well as any relevant public health agency or authority. A custom-made mobile app was used to collect big data from participating children and adolescents in 5 different European countries (see the *Data* section); the extracted information has been visualized in the OPdashboard. In brief, the functionality includes: (1) real-time monitoring and visualization of children’s behavior, (2) extraction of associations between behavior and environment in the areas close to schools, (3) evaluation of interventions as well as (4) the design of interventions using behavioral prediction models. Each of the functionalities are described in more detail in the *OPdashboard Functionality* section. In the *Results* section, the effect of the living environment close to schools on children’s behavior is described.

Lastly, we also explored the impact of COVID-19. The emergence of the COVID-19 pandemic resulted in the adoption of measures, at a national level, to decelerate disease spread in the population. Those interventions varied across countries, ranging from social distancing recommendations to partial or total lockdown. Data analysis based on wearable activity trackers has shown that home confinement policies affected physical activity levels in the general population [24]. An evaluation of these measures, in terms of behavioral changes associated with characteristics of the local environment, may reveal additional information to health authorities.

In addition, the effect of COVID-19–related measures implemented in Greece on the children’s behavior is presented in the *Results* section. The evaluation of the OPdashboard’s functionality and usefulness was performed by experts in the field of childhood obesity and public health policy, as described in the *Evaluation* section. The OPdashboard will be made freely available to the scientific community via a web interface after the conclusion of the BigO program. The expected impact of the OPdashboard, as well as its limitations, are discussed in the final section.

## Methods

### Data

Within BigO, the cornerstone of the data collection process is *citizen science,* which is a relatively new scientific approach to gather big data, where the general population actively contributes to scientific research [25]. In that vein, school students, aged 9 to 18 years, were recruited and following an informed parental consent process, agreed to participate in data collection as part of citizen science projects, using a custom mHealth (mobile health) app available for both Android and iOS devices [26]. Several questions related to children’s daily mood and sleeping behavior are asked through the myBigO app. In addition, the app allows the users to submit photos related to eating behavior. Awards, in terms of virtual badges, are provided to frequent users in order to maximize adherence to data collection. Furthermore, objectively collected GPS and accelerometer data, via the myBigO app, were analyzed in order to extract aggregated population-level obesogenic behavioral indicators identified within a specific geographical region [20]. The geohash geocoding system [27] was adopted as a spatial structure describing a broader geographical area, the size of which varies according to the length of the codes considered. In the OPdashboard, geohashes of 6 and 7 digits were used to reflect an area of 1.22 km × 610 m and 153 m × 153 m on the equator, respectively, with a reduction in width the further away from the equator. The aggregation to geohash level was done as a privacy preservation measure in order to avoid potential subject identification from GPS data.

Per Diou et al [20], personal sensory data, such as GPS and accelerometry, were used to extract individual indicators. The aggregated behaviors were calculated either based on the unique individuals visiting a geohash, or based on all values across the visits, even if they are visits from the same individual, while various geohash sizes are supported through the OPdashboard. Furthermore, aggregations are also performed based on the individuals living in a geohash. The rationale behind this approach is the “residential fallacy” effect [22], which underlines the importance of the nonresidential places visited in order to estimate residential effects.

In order to have a more reliable estimation of a behavior in a geohash area, the aggregation was performed only in case more than 5 values were recorded there (ie, from unique visits or unique visitors, respectively). All data were transmitted with end-to-end encryption and stored on secure servers. The stored data did not contain any directly identifiable information (eg, names or emails).

In keeping with co-design principles, a Delphi panel was carried out with public health experts across Europe with a remit in obesity prevention in order to prioritize the elements of interest (behavior and environmental and socioeconomic parameters) that should be measured and visualized as part of the OPdashboard [28]. Proposals were then assessed based on the feasibility of the measurements during the BigO project, and finally 38 behavior indicators, 40 environmental parameters, and 6 socioeconomic parameters were defined.

Behavior indicators were distinguished into two main categories. The first category was related to eating behavior and addressed food consumption and frequency of visitations to food-related locations, whereas the second category was related to physical activity behavior and addressed the frequency of visits to sports-related places and measurements of physical activity, such as number of steps per day. More information related to the methodology adopted for the extraction of the behavioral indicators based on the raw accelerometry and geolocation data can be found in Papapanagiotou et al [29]. Table 1 provides a subset of the behavioral indicators that have been calculated in the context of BigO. Through the OPdashboard, only aggregated behaviors are presented and no individual data are available.

Similarly, 40 environment characteristics of interest were identified and included in the BigO system (Table 2). These broadly describe the availability of diverse type of places in a geographical region of a geohash and were collected through open and online data sources [20].

Finally, the socioeconomic characteristics of the local area were also highlighted by public health obesity experts as being crucial for the evaluation of childhood obesity [13]. In this respect, taking also into account the availability of the data in a regional level from national databases and Eurostat, a number of socioeconomic factors were also considered. However, this information is not available at the geohash level and was thus measured in the broader geographical area of a municipality.

**Table 1:** A subset of the behaviors used in BigO.

Behavior	How it was computed
Average steps per hour across visits to the region	For each visit to the region, take the total number of steps during the visit, as well as the duration of the visit and compute the average steps/hour. This behavior is the average of these values across visits (even if they are visits from the same individual). For the computation of this indicator, only visits that last 1 minute or more are considered.
Average steps per hour across visitors of the region	For each unique visitor of the region, take the total number of steps during his or her visits, as well as the total time he or she spent in the region, and compute the average steps/hour. This indicator is the average of these values across unique visitors. For the computation of this indicator, only visits that last 1 minute or more are considered.
Percentage of visits to the region that include at least one visit to a food-related location	Compute the percentage of visits to the region that include at least one visit to a food-related place. For the computation of this indicator, only visits that last 1 minute or more are considered.
Percentage of visits that include at least one visit to an athletics or sports facility	For each visit to the region, take the number of visits to an athletics or sports facility. This indicator is the average across visits. For the computation of this indicator, only visits that last 10 minutes or more are considered.
Average daily number of steps for residents of the region	For each resident of the region, compute his or her steps using the recorded data across all the areas that the resident visited. Based on this, compute his or her daily average number of steps for days with more than 60 minutes of recorded data (days with fewer data points are discarded). This indicator is the average of this value across the residents of the region but corresponds to the behaviors that might have happened anywhere on the map.

**Table 2:** A subset of the environmental characteristics used in BigO.

Environmental characteristic	How it was computed
Number of athletics or sports facilities in the region	Using publicly available data sources, compute the number of athletics or sports facilities in the region.
Number of fast food outlets in the region	Using publicly available data sources, compute the number of fast food outlets in the region.
Average number of restaurants within a 100-meter radius from locations within the region	Create a 30-meter point grid inside the region. For each point, compute the number of restaurants within a 100-meter radius. This value is the average across all points inside the region.

### OPdashboard

The OPdashboard is a web application for public health scientists and policy makers, which supports decision making and planning of localized interventions to reduce the prevalence of childhood obesity. The OPdashboard is freely available and the beta version can be accessed online [30].

#### Requirements

Methodologically, the development of the OPdashboard has taken jointly into account (1) expert-driven predevelopment requirements and (2) mid-development expert evaluation with regards to relevance within the childhood obesity and public health domains.

Based on public health experts’ knowledge, a set of mock-ups were designed. In order to ensure that the specific functionality of the OPdashboard meets the practical needs of public health authorities, focus groups and interviews with experts in nutrition, obesity, behavior, and public health were arranged to demonstrate and elicit feedback on the proposed functionality of the system. Based on the presented methodology and through systematic evaluation of the collected feedback, a list of requirements were defined reflecting both the functionality requirements and requirements relevant to the developed interface design.

According to the feedback from the focus groups, the following functionalities were recommended for integration into the OPdashboard:

- Explore: description of aggregated local data on children’s behaviors, socioeconomics, and environment;
- Explain: visual comparisons between behaviors and the environment in a given area, as well as the extraction of associations;
- Compare: comparison of behaviors across time, which can be used to monitor and evaluate an intervention;
- Predict: simulation of behavioral change upon changes in an environment characteristic.

The first version of the OPdashboard focused on the first three of these functionalities. In the future, it is anticipated that predictive models of high accuracy will be developed based on the ongoing big data collection, supporting the design of future interventions.

#### Technical Implementation

All the data required by the OPdashboard are stored in a Mongo database (MongoDB), which is a cross-platform document-oriented database management system. A number of RESTful web services [31] have been developed using the Jersey framework [32]. These services access the MongoDB with the help of the MongoDB Java Driver [33], using a Mongo account defined specifically for the service that is granted with the proper set of permissions (only read/select access on specific Mongo collections). The endpoints offered by the service require authorization via a valid, BigO-generated, JSON (JavaScript Object Notation) web token (JWT) [34].

The interactive web application was implemented using the R Studio Shiny server [35]. Shiny allows simple HTML pages to interactively execute R scripts and takes advantage of R’s visualizing capabilities to graphically present the analysis results on the webpage. In order to consolidate the user experience, Shiny applications can be further extended by using JavaScript and jQuery. The R-script execution and the use of the analysis and visualization mechanisms inside the HTML pages are controlled via the user interface–rendering engine of the Shiny web server.

#### Extraction of Associations

Regarding the extraction of associations between the behaviors and the environment, a number of studies propose that the proximity of fast food outlets to schools may have an effect on adolescent eating patterns and may contribute to obesity [15,36]. In this respect, the analysis was based on the extraction of associations between the population behavior and the environment close to schools. For the definition of this area, only the geohashes where the distance between the center of the geohash and the location of the school is less than 1000 m were considered. For this analysis, a 7-digit geohash was used.

For these areas, each behavior detected within a geohash area was correlated with the environmental characteristics of the respective geohash in order to investigate the existence of a correlation between them. The Pearson correlation coefficient was computed which is defined as:


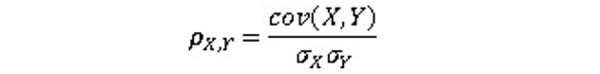


where *X* and *Y* denote the 2 variables, *cov* is the covariance, and σ the standard deviation of a variable. The *P* values for the estimation of the statistical significance of the correlation coefficients are also computed. The linear correlation analysis is made available on the dashboard in order to provide a preliminary understanding of the data.

It is worthy to mention that cultural and ethnic backgrounds also have the potential to affect users’ behavior and their association with the environment [37], especially considering the case of BigO where data are distributed across different countries. In order to minimize the effect of possible confounders that can bias the results, the analysis was performed separately for each city.

#### Comparison in Time: The COVID-19 Paradigm

The compare functionality of the OPdashboard allows for the comparison of obesogenic behaviors in different time frames. These time frames represent the period before and after interventions that focus on the modification of obesogenic behaviors or exposure to obesogenic environments to support a healthier lifestyle. Due to the lack of typical obesity-related interventions in the before period, mainly because of the COVID-19 pandemic, the paradigm presented here was based on a scenario that monitored children’s behavioral change as a result of the nation-wide measures undertaken to limit the spread of COVID-19 in Greece [38].

Greece adopted strict measures to restrain the pandemic, leading to the closure of schools on March 11, 2020, while a lockdown was implemented on March 23. Since the goal of the analysis was to investigate the effect of the adopted policies on children’s behavior, the analysis was carried out on the Greek population, and in the city of Thessaloniki in particular, where the majority of the data were collected. The target behavior was steps per hour since visits to specific places were prohibited during the lockdown and only outdoor activity was allowed for all citizens.

The analysis was made for all 8 municipalities of Thessaloniki, while the behavior for each municipality was calculated as the aggregation of all the geohashes within the boundaries of each municipality where the behavior from at least one user was identified. For this analysis, 7-digit geohashes were considered.

In this respect, the aggregated steps per hour in each municipality of Thessaloniki, before and after school closure, were compared. The period before was defined as ranging from January 8, 2020, when schools in Greece opened after the Christmas holidays, to March 10, 2020. The after period was defined as March 11-31, 2020. Recruitment was low after this date, and data for the post–March 31 period was not considered in the analysis. The two-sample *t* test was used to investigate the existence of statistically significant differences in behavior across time.

#### Predictive Models

The OPdashboard allows for the downloading of the aggregated behaviors at the geohash level of detail. This functionality offers the possibility to further analyze the data and to implement models that are able to predict the behavior of the population based on the environment. Initial works carried out by the BigO team have used such data in order to predict children’s physical activity, achieving a prediction accuracy of 81% [39]. However, the implementation of such models as well as the investigation of their accuracy is beyond the scope of this work. Nonetheless, such models have been integrated in the OPdashboard, offering a visual interpretation of behavior in a city when no behavioral data are available in specific regions.

In addition, the OPdashboard offers the possibility to design specific interventions in a region and predict their effect on certain obesogenic behaviors in children. The design of the interventions can be made graphically by modifying the characteristics of the environment (eg, adding or removing public parks in a region), while the effect of the intervention can be assessed using the map view.

#### OPdashboard Evaluation

The OPdashboard was demonstrated to a wide audience during the European Childhood Obesity Group Conference while a focus group comprising experts in public health, statistical epidemiology, and clinical endocrinology was organized in order to obtain feedback on the dashboard’s usability.

After the live demonstration of the OPdashboard, the following questions were posed to the experts:

- In your opinion, what is the value of the BigO system for local population behavioral assessment?
- In your opinion, what is the value of the BigO system in discovering associations between localized behaviors and environments? What additional steps would be necessary to discover causal relationships?
- In your opinion, which users/public authorities would most benefit from BigO? (eg, epidemiologists, population health consultants, local governments, educational policy makers)
- In your opinion, what are the main barriers for the adoption of BigO by public health authorities?

This discussion was recorded using a microphone, and a report was produced. The outcome of this focus group helped define the improvements needed and are described in the *Evaluation* section.

## Results

### Data Overview

The OPdashboard constitutes a live system, where more and more schools are added over time. By April 2020, BigO was deployed in 33 schools and 2 pediatric clinics in 5 different European cities (Athens, Thessaloniki, and Larissa from Greece; Stockholm from Sweden; and Dublin from Ireland) [40]. Ethical approval was received from each country separately. Each child was asked to use the myBigO app for at least 2 weeks. A steady flow of recruitment was followed; however, an expected decrease occurred during the summer because of the summer holidays and school closure. Most of the children used the app during the 2-week period, while a small minority continued to use it for more than 2 weeks

More than 3700 children, aged 9 to 18 years, participated in data collection, between the start of March 2018 until the end of March 2020, providing approximately 107 years of accelerometry data and 73 years of GPS data [40]. This was the first time such an amount of big data has been collected contemporaneously, on an individual basis within a region, allowing for the extraction of childhood obesogenic behavior accurately. The majority of the schools included in the analysis are located in the city of Thessaloniki, Greece, and Stockholm, Sweden, and thus the analysis in the following sections will be based on the schools from these cities only, as seen in Table 3.

**Table 3:** Details on the collected data from Thessaloniki, Greece, and Stockholm, Sweden.

Characteristic		Location	
		Thessaloniki	Stockholm
Children, n		839	671
Age (years), mean (SD)		13.5 (2.2)	15.3 (1.8)
**Sex, n**			
	Male	454	375
	Female	380	265
	Not available	5	31
Z-score >1 (%)		26.1	18.3
Schools, n		24	5
Accelerometer (hours)		179,969.48	78,903.17
GPS (hours)		112,624.13	225,337.67

### OPdashboard Functionality

In this section, the current version of the OPdashboard is presented. It focused on the data exploration (“explore”) and the extraction of associations (“explain”) between children’s behavior and the environment close to schools. Regarding the “compare” functionality, the analysis focused on the comparison of the population’s steps per hour before and after the implementation of national health policies due to COVID-19. The “predict” functionality was not presented here for the reasons made clear earlier.

#### Data Exploration Overview

**Figure 1:**
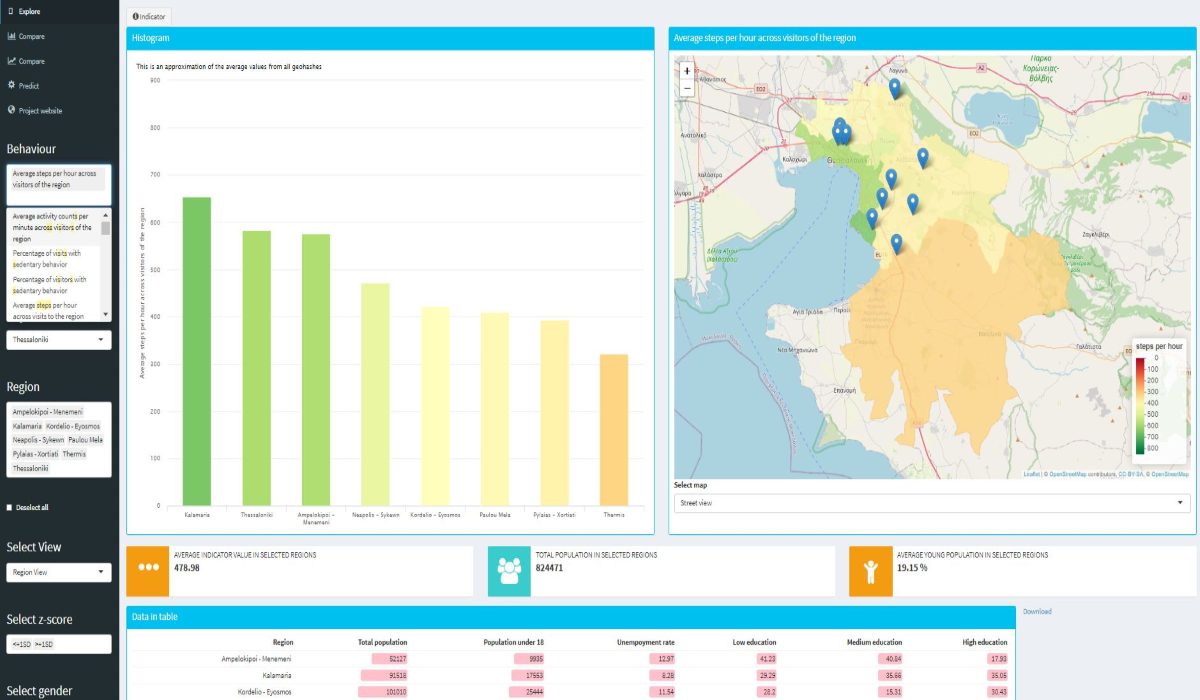
The OPdashboard main page with the control panel (left) and the main panel (right).

The main screen of the web interface is shown in Figure 1. It is divided into two main areas, one focusing on the selection of the variables to be analyzed (control panel), whereas in the second area the results are visualized (main panel). The available selections include behavior and environmental characteristics as well as the city of interest, in terms of the broader metropolitan area, which can be further divided into the level of available municipalities.

Furthermore, the user can select the representation level, including the region view, where the population-aggregated value of the variable is depicted for the selected municipalities of the city, and the geohash view, where the aggregation is made at the geohash level. Filters related to the BMI z-score and gender can also be applied in order to focus on specific groups of children. Finally, a list of regional socioeconomic information related to (1) the total population, (2) their educational level, (3) the percentage of young people, and (4) the unemployment rate is also provided in a tabular format.

At the top of the analysis panel, the 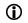 button serves as a tool to provide additional information regarding the selected behavior or environment characteristic, highlighting its importance as well as describing how it was measured.

The dashboards are provided within the analysis panel, allowing the user to have a clear overview of the indicators and their distribution across the regions. For data exploration, both bar charts and map views are provided to the user. A tabular representation of the socioeconomic data for the selected regions is provided as this information can be considered as a confounder contributing to a specific behavior or environmental characteristic.

The bar charts provide in a sorted format the aggregated value in each municipality of the city. This comprehensive visualization allows for the direct comparison of different variables across several municipalities of the city, as well as between each municipality and the average value across the selected regions.

**Figure 2:**
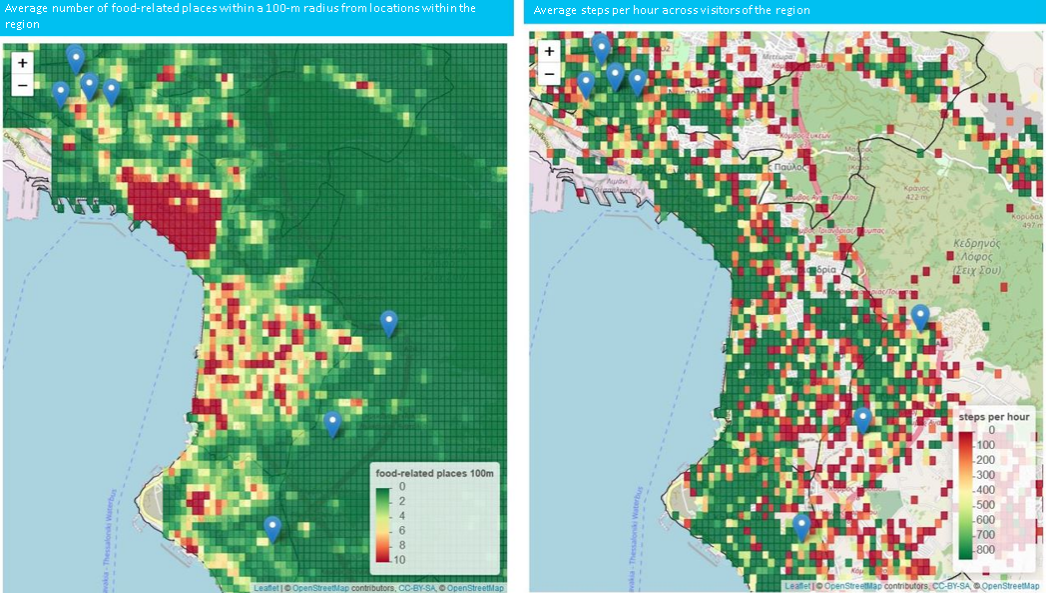
Geohash distribution of food-related locations within a 100-meter radius from each geohash (left) and the steps per hour (right) for the city of Thessaloniki. Using mouseover, the user can access more information related to the number of contributors and the actual value in each geohash. Note: the geohash is visible only in cases where the aggregation was based on more than 5 contributions. The visual exploration reveals the high density of food-related places in the downtown of Thessaloniki and high activity among the children near the coastline of the city. The blue pins denote the location of schools. Source: OpenStreetMap [41].

The map view allows user to quantify the value of each variable within a geographical area. This information can be depicted as an aggregation of the behavior across the municipality, or it can be represented in more detail using the geohash visualization (Figure 2). Blue pins on the map highlight the location of the schools that have been included in the BigO pilots.

**Figure 3:**
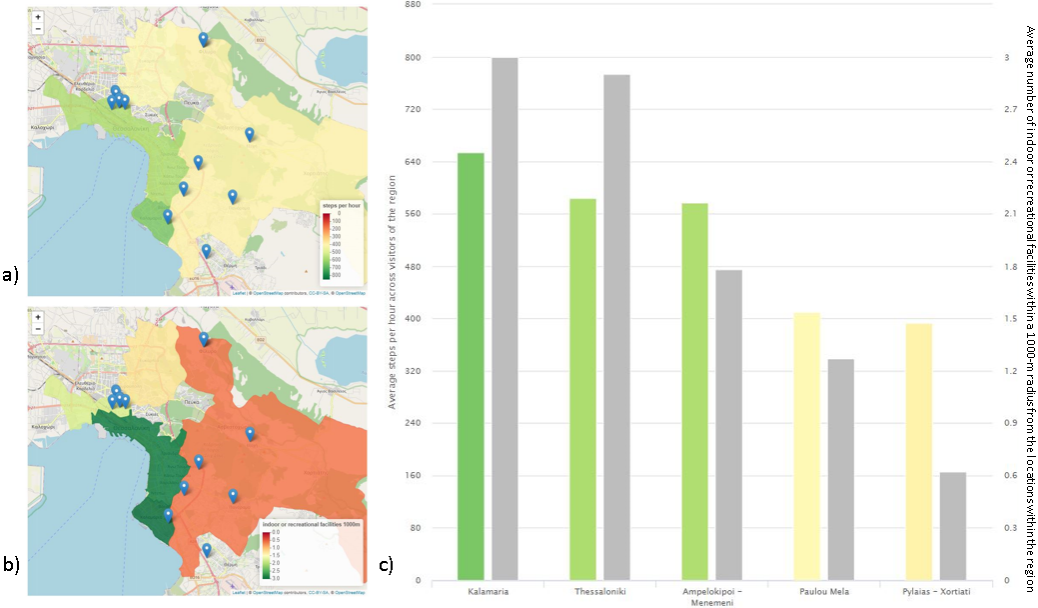
Regional representation of the steps per hour in the regions where schools are located (A) and the number of indoor recreational or sports facilities in those areas (B). The visual comparison between the two variables reveals an association between them (C). In (C), the color of the second indicator is set to gray in order to avoid any confusion to the user.

The coloring format used in the aforementioned visualizations ranges from red to green or vice versa, according to the type of indicator. In other words, the coloring changes from red to green as the number of steps per hour increases. On the other hand, when the small value represents a healthy state (eg, low number of visits to fast food restaurants, low number of food outlets in the region), then the coloring changes from green to red to indicate an increase in the number of food outlets in the region. The maximum value for each variable was extracted either by a review of the literature, or derived as the 95th percentile based on the collected data. In addition, the OPdashboard allows for a visual comparison between behaviors and/or the environment through bar charts (Figure 3). The user can extract the data, in a JSON format, in order to perform further analysis.

The visualization of a behavior at the geohash level of detail was made only for those geohashes where a behavior was observed.

#### Associations Between the Environment and Behaviors Close to Schools

**Figure 4:**
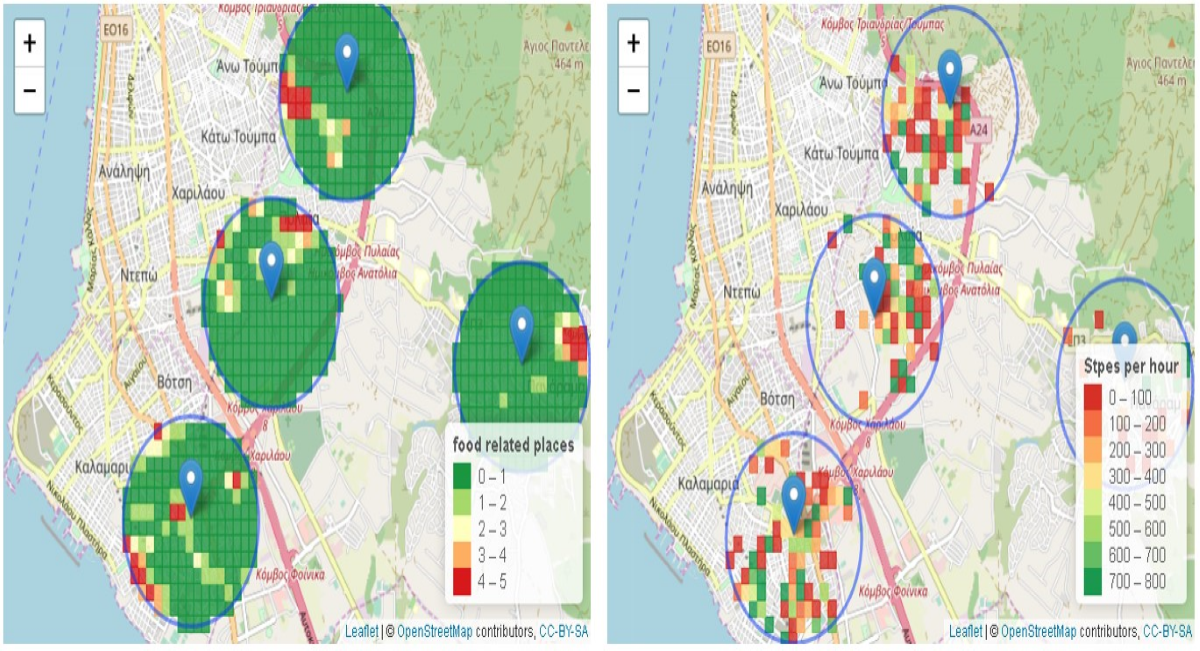
An area of Thessaloniki where 4 schools are located (blue pins). The blue circles (1000-meter radius) define the regions under analysis close to schools. In the right image, the steps per hour for each geohash are depicted, whereas the density of food-related places for the same region is depicted on the left. Visual observation of the images revealed that there should exist a correlation between the density of food-related places close to schools and the steps per hour performed by the children in those areas. Source: OpenStreetMap [41].

The analysis performed here was based on the data collected in Thessaloniki and Stockholm, where the majority of schools are located. In order to increase the reliability of the results, only the geohashes visited by more than 5 unique users or visited more than 5 times were analyzed. Figure 4 provides a visual representation of the behaviors identified close to schools and the respective environment in the city of Thessaloniki. Through the OPdashboard, the user can select a subset of schools in order to focus the analysis on a specific population.

In total, 12 behavioral indicators were analyzed. Those behaviors were categorized into two groups—the first one reflects the activity (eg, steps/minute or % of sedentary behavior) while the second reflects the visits to a specific place (eg, % of visits to fast food places). For the first group of behaviors, the associations were computed based on environmental characteristics either reflecting the availability of food stores or sports-related facilities. For the second group, the analysis was focused on the extraction of associations between the behavior and the density of the respective places (eg, % of fast food or takeaway visits with the density of fast food or takeaway shops in the geohash).

The OPdashboard offers automated analysis for Stockholm and Thessaloniki. The results of the Pearson correlation analysis are provided in Table 4. The results for Thessaloniki reveal the existence of statistically significant correlations between physical activity levels and the availability of food stores and sports facilities close to schools—that the higher the density of these shops or facilities in an area, the greater the chances a child will visit them (Table 4). In other words, in Thessaloniki, the use of resources in the areas close to schools was high. In order to explain this difference, additional confounders must be taken into account, such as the total population in these areas, the age of the children, and psychological factors [16].

**Table 4:** Correlation analysis for each behavior and the most relevant environmental characteristic within a 1-km radius from a school for students in Thessaloniki (24 schools). “Food” and “Sports” are computed as the summation of all relevant environmental characteristics reflecting the availability of food places or sports-related facilities. “Visitors” indicate the aggregation of behaviors from unique children visiting a geohash, while “visits” indicate aggregation from all the visits to a geohash even when performed by the same children. For the areas close to schools, 281 geohashes with more than 5 unique visitors and 523 geohashes with more than 5 visits were analyzed.

Behavior and environment		Thessaloniki	
		Correlation (CI)	*P* value
**Steps/hour (visitors)**			
	Food	0.123 (0.006-0.237)	.04
	Sports	0.128 (0.011-0.241)	.03
**Steps/hour (visits)**			
	Food	0.129 (0.043-0.212)	.003
	Sports	0.141 (0.056-0.224)	.001
**% visits to**			
	Food places	0.221 (0.138-0.301)	<.001
	Fast food or takeaway places	0.302 (0.222-0.378)	<.001
	Supermarkets or grocery stores	0.209 (0.211-0.368)	<.001
	Athletics or sports facilities	0.325 (0.246-0.400)	<.001
	Indoor recreation facilities	0.305 (0.225-0.381)	<.001
	Public parks	0.248 (0.166-0.327)	<.001

#### Comparison of Children’s Activity in Time

**Figure 5:**
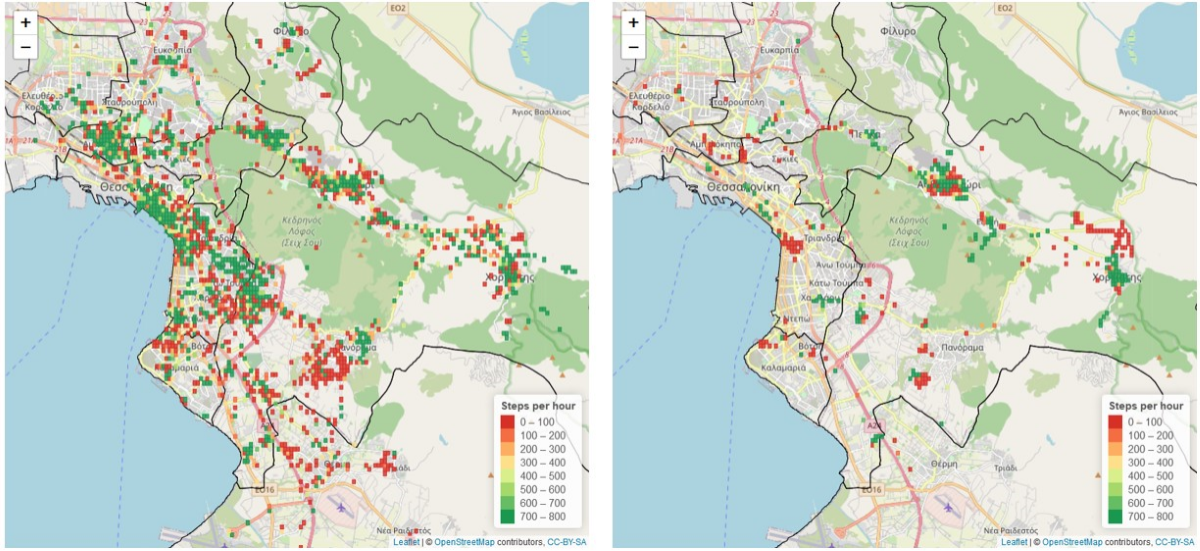
Effect of school closure due to COVID-19 on children’s physical activity levels. The steps per hour for the period between January 8, 2020 (opening of schools after the Christmas holidays) and March 10, 2020 (school closure) are depicted on the left, while on the right, the steps per hour for the period between March 11 and 31 (end of data collection) is depicted. The black lines define the boundaries of each municipality in the city of Thessaloniki. Source: OpenStreetMap [41].

For the period before school closures (January 8 to March 10, 2020), 1802 geohashes were analyzed where at least one visit by a child was performed and a behavior was detected (Figure 5). On the other hand, in the period between March 11-31, 2020, 427 geohashes were analyzed. This expected decrease in the number of geohashes is attributed to the restrictions on population mobility as the result of the COVID-19 pandemic. A decrease in the average steps per hour was detected for the visitors of 6 municipalities within the Thessaloniki metropolitan area, while on the other hand, a slight increase was found for 2 municipalities. In particular, for the municipalities of Ampelokipoi-Menemeni and Kordelio-Evosmos, a statistically significant reduction in steps per hour was observed. Upon closer analysis, one can observe that the municipalities where an increase in physical activity was observed were suburban areas with ample open spaces. This observation implies that school closures provided an opportunity for children to exercise outside. On the contrary, the highest decrease was observed in the westside regions, possibly due to the lack of open spaces.

Finally, statistically significant differences were also observed in the downtown area of the metropolitan part of Thessaloniki, covering the area close to the coast. Under normal conditions, children visit the coast to spend their free time; however, after the closing of schools and probably due to the fear of COVID-19, children ceased visiting this area, as shown in Table 5.

**Table 5:** Effect of school closures on the aggregated number of steps per hour in Thessaloniki as a measure to restrain the COVID-19 pandemic.

Municipality	Before		After		*P* value
	Geohashes, n	Average steps per hour	Geohashes, n	Average steps per hour	
Thermis	132	163.09	7	206.07	.74
Pylaia-Chortiatis	793	379.32	275	401.90	.37
Kalamaria	81	304.20	15	160.85	.09
Thessaloniki	421	468.90	60	358.62	.03
Ampelokipoi-Menemeni	87	561.37	14	46.45	<.001
Neapoli-Sykies	138	484.42	24	388.25	.22
Kordelio-Evosmos	45	516.75	11	226.35	.02
Paulou Mela	105	434.89	21	415.46	.81
Total	1802	404.54	427	367.86	.06

### Evaluation

The evaluation of the first version of the OPdashboard regarding its usability and prospect of use, mainly focusing on data exploration, was performed in a focus group that took place in Katowice, Poland, on November 14, 2019, as a parallel session of the ongoing meeting of the European Children Obesity Group. During the focus group, a live demonstration of the OPdashboard was performed, and no technical issues arose.

The focus group participants mentioned the existence of many applications, software, and geographical information systems (GIS) that take data from national authorities or Eurostat and present them in map views and dashboards; however, it was noted that there was nothing available with this type of behavioral data from children at the local level and in such detail. The integration of these data with nationally collected statistics was considered by the participants to be a major strength of the OPdashboard.

In addition, during the focus group, it was mentioned that the OPdashboard potentially offers a very powerful interface with a dashboard and maps, providing insightful information on local data. This visual tool could be very useful to local health authorities to explore localized childhood behaviors and big effects. The OPdashboard therefore offers a contribution to science and public health (and clinical management) as well as the collection and use of big data in finding solutions to real-life problems.

Furthermore, regarding the analysis of the available data, it was highlighted that the analysis must focus on local use and avoid comparisons across countries. The main reason mentioned was a lack of standardization in the classifications of environmental parameters when using data from various countries to look at relationships. A large number of confounders must also be considered in order to extract causal relations between obesogenic behaviors and the environment.

One additional aspect that may affect the analysis is the estimation of the lack of standardization for the characterization of the environment. In the BigO system, environmental parameters such as points of interest use the data from GIS and online data sources, such as Google Maps, Foursquare, and OpenStreetMap. However, there are big differences in this terminology between countries. For example, fast food stores offer different types of foods in Greece (healthy foods can also be available in Greek fast food stores) and Sweden. If international comparisons are made, the analysis that is made through the OPdashboard must take into account the heterogeneity of classifications in environmental variables.

The OPdashboard can be used for the evaluation of interventions, through analysis made longitudinally with time, or for the design of health policies through the application of predictive models. In this respect, during the focus group, it was mentioned that the users who would benefit most from the OPdashboard will be local authorities, central governments, educational policy makers, and researchers (eg, epidemiologists) since these user groups play an important role in the development of effective interventions or actions to address local childhood obesity–related issues. Finally, engaged citizens may also find the system valuable due to high interest in exploring data from their region and country.

It was identified that confidentiality and privacy of data may be the main barrier against the adoption of the OPdashboard, and BigO in general, by health authorities.

In conclusion, it was underlined that BigO, and the OPdashboard in particular, can be very helpful to public health authorities for planning interventions and observing their effect in the local population.

## Discussion

### Principal Findings

The increased prevalence of obesity and overweight in children in European countries has been identified as a major public health issue that must be addressed. Child and adolescent obesity can occur into adulthood and is associated with increased morbidity and mortality. Modern technologies and their widespread adoption by children and adolescents can facilitate a better understanding of children’s obesogenic behavior, particularly those that increase the likelihood of developing obesity early in life. In addition, the characteristics of the environment in which children move, eat, and live can promote obesogenic behaviors, and these must be considered in order to design effective localized interventions at the population level.

In this paper, we presented the OPdashboard, which is an interactive tool that may be used for the extraction of associations between children’s obesogenic behaviors and the local environment using big data. The OPdashboard is freely available and the beta version can be accessed online [30]. The main functionalities of the OPdashboard include data exploration, extraction of associations, evaluation of interventions, and prediction of a behavior after an intervention. The OPdashboard was implemented as a web interface for potential users like (1) local health authorities and policy makers who develop and deliver actions to change obesogenic factors and reduce obesity, (2) researchers focusing on understanding obesity and the factors that influence it (eg, data scientists, epidemiologists), and (3) public and local educational and social services. In addition, citizens who are interested in exploring the data of their region and/or country can also benefit from the provided tools. The OPdashboard was implemented in the context of the BigO program [23], which aims to tackle the problem of childhood obesity.

Over 3700 children from more than 33 schools and 2 clinics in 5 European cities used a custom-made mHealth app, through which accelerometry and geolocation data were recorded. The big data collected led to the extraction of population behaviors in specific geographical areas while online databases were used for the collection of data related to the characteristics of the environment.

The web interface of the OPdashboard was implemented in R while RESTful web services were developed in order to access data stored in the MongoDB database. The data exploration functionality was evaluated by a focus group, where experts in the field of public health, statistical epidemiology, and clinical endocrinology participated, highlighting the usefulness of such a tool for the understanding of obesity in association with the environment.

Our findings indicated that the greater the density of food- or sports-related places in the areas proximal to schools, the greater the probability that children will visit them. These results highlight the effect of the local environmental factors on children’s behavior, as previously documented in several studies [15,16].

The outbreak of the COVID-19 pandemic triggered an assessment of children’s behavioral change as a result of the health-related policies implemented. In this respect, the effect of school closure in Greece on children’s physical activity was studied. It was found that school closure led to an expected decrease in children’s physical activity in the city of Thessaloniki. However, the decrease was statistically significant in urban areas, while in the suburbs, the decrease was not so apparent. On the contrary, in some municipalities, an increase in children’s physical activity levels was observed. This finding might be the result of a different mix of underlying socioeconomic, cultural, and environmental factors related to obesogenic behaviors among children that was not addressed in the design of this study.

A recent systematic review on the use of telehealth for the treatment of childhood obesity was shown to be promising, particularly for reaching rural and less accessible patients, and carefully designed mHealth interventions have the potential for improving this reach, given the increasing popularity of mobile devices [42]. However, the prevention of obesity, or nutritional disorders in general, using mHealth apps is still under development, with some studies evaluating the feasibility of using such apps for the prevention of nutritional disorders using food intake metrics, activity level metrics, and questionnaires [43]. The impact of preventive interventions through advanced mHealth systems is expected to increase due to a lack of large-scale databases, a gap that our OPdashboard system tries to close. Furthermore, given that pediatricians and other health professionals involved in the management of childhood obesity appear inclined to incorporate mHealth systems into their practice [44], we expect the OPdashboard to be adopted by the medical community as is the case with the educational community.

The unique big data that are available through the OPdashboard can lead to the implementation of models that are able to predict population behavior, allowing for the design of localized interventions. The OPdashboard can be considered as a tool that will increase our understanding of the underlying factors in childhood obesity and the design of regional interventions both for prevention and treatment.

### Limitations

Limitations include problems regarding data sparseness such as missing data; lack of continuous monitoring at the individual level; and variations in the accuracy of the estimates, which may lead to bias or inaccurate correlation estimates. Overcoming these limitations is in progress.

There is also an underestimation of daily physical activity as the dashboard relies on smartphone-based accelerometry measurements, and smartphones are not carried continuously by children. Sports activities are usually missed. In some countries such as Greece, students may not be allowed to use their smartphones in school, further limiting the collection of accelerometry data during the day.

Furthermore, to ensure completeness of the analysis, additional confounders affecting children’s behaviors must be studied, which were not considered here. These cofounders include, among others, psychological, socioeconomical, or cultural factors. Additionally, the age, gender, and BMI z-score of the children was not considered in the analysis. An analysis of the type of meals based on photos taken by the children using the myBigO app could provide more insights on children’s obesogenic behaviors; however, this was out of the scope of this study.

Finally, another limitation of the study was the high administrative load due to the exclusive use of paper-based parental consent for children’s participation, which resulted in low participant numbers in some schools. Following the decision of the ethics review boards in Greece and Sweden, electronic consent via the mHealth app, which could facilitate user registration, was deemed inappropriate due to the young age of the participants and the sensitive nature of the collected data.

## Abbreviations

GIS: geographical information system
JSON: JavaScript Object Notation
mHealth: mobile health
OPdashboard: Obesity Prevention dashboard
